# (2*E*)-2-(3-Eth­oxy-2-hy­droxy­benzyl­idene)hydrazinecarboxamide

**DOI:** 10.1107/S1600536813007617

**Published:** 2013-03-23

**Authors:** A. Ambili Aravindakshan, M. Sithambaresan, M. R. Prathapachandra Kurup

**Affiliations:** a Department of Applied Chemistry, Cochin University of Science and Technology, Kochi 682 022, India; b Department of Chemistry, Faculty of Science, Eastern University, Sri Lanka, Chenkalady, Sri Lanka

## Abstract

The title compound, C_10_H_13_N_3_O_3_, adopts an *E* conformation with respect to the azomethine bond and crystallizes in the amide form. A classical intra­molecular O—H⋯N hydrogen bond is present. The two N atoms of the hydrazinecarboxamide unit are also involved in inter­molecular N—H⋯O hydrogen bonds, with the O atom of the hydrazinecarboxamide group acting as the acceptor. Pairs of N—H⋯O hydrogen bond link the mol­ecules into centrosymmetric dimers, which are linked by further N—H⋯O hydrogen bonds into chains along the *b* axis. The chains are linked by C—H⋯π inter­actions.

## Related literature

For biological applications of hydrazinecarboxamide and its derivatives, see: Afrasiabi *et al.* (2005 ▶); Siji *et al.* (2010 ▶); Beraldo & Gambino (2004 ▶). For related structures and background references, see: Sithambaresan & Kurup (2011 ▶); Noblía *et al.* (2004, ▶ 2005 ▶); Benítez *et al.* (2009 ▶, 2011 ▶); Rivadeneira *et al.* (2009 ▶); Gambino *et al.* (2011 ▶). For standard bond-length data, see: Allen *et al.* (1987 ▶); Kala *et al.* (2007 ▶). For the synthesis, see: Sreekanth *et al.* (2004 ▶).
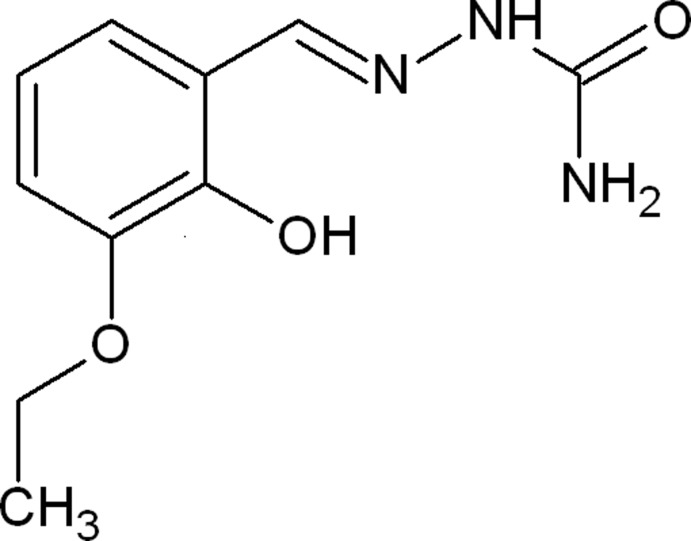


## Experimental

### 

#### Crystal data

C_10_H_13_N_3_O_3_
*M* *_r_* = 223.23
Triclinic, 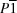

*a* = 5.0676 (4) Å
*b* = 7.0426 (7) Å
*c* = 15.8394 (15) Å
α = 97.509 (4)°
β = 98.819 (3)°
γ = 105.790 (4)°
*V* = 528.62 (8) Å^3^
*Z* = 2
Mo *K*α radiation
μ = 0.11 mm^−1^
*T* = 296 K
0.30 × 0.25 × 0.20 mm

#### Data collection

Bruker Kappa APEXII CCD diffractometer
Absorption correction: multi-scan (*SADABS*; Bruker, 2004 ▶) *T*_min_ = 0.969, *T*_max_ = 0.979
2559 measured reflections
1794 independent reflections
1496 reflections with *I* > 2σ(*I*)
*R*_int_ = 0.011

#### Refinement

*R*[*F* ^2^ > 2σ(*F* ^2^)] = 0.037
*wR*(*F* ^2^) = 0.109
*S* = 1.05
1794 reflections
163 parameters
5 restraints
H atoms treated by a mixture of independent and constrained refinement
Δρ_max_ = 0.15 e Å^−3^
Δρ_min_ = −0.18 e Å^−3^

### 

Data collection: *APEX2* (Bruker, 2004 ▶); cell refinement: *APEX2* and *SAINT* (Bruker, 2004 ▶); data reduction: *SAINT* and *XPREP* (Bruker, 2004 ▶); program(s) used to solve structure: *SHELXS97* (Sheldrick, 2008 ▶); program(s) used to refine structure: *SHELXL97* (Sheldrick, 2008 ▶); molecular graphics: *ORTEP-3 for Windows* (Farrugia, 2012 ▶) and *DIAMOND* (Brandenburg, 2010 ▶); software used to prepare material for publication: *SHELXL97* and *publCIF* (Westrip, 2010 ▶).

## Supplementary Material

Crystal structure: contains datablock(s) I, global. DOI: 10.1107/S1600536813007617/fj2621sup1.cif

Structure factors: contains datablock(s) I. DOI: 10.1107/S1600536813007617/fj2621Isup2.hkl

Supplementary material file. DOI: 10.1107/S1600536813007617/fj2621Isup3.cml

Additional supplementary materials:  crystallographic information; 3D view; checkCIF report

## Figures and Tables

**Table 1:** Hydrogen-bond geometry (Å, °) *Cg* is the centroid of the C1–C6 ring

*D*—H⋯*A*	*D*—H	H⋯*A*	*D*⋯*A*	*D*—H⋯*A*
N3—H3*A*⋯O3^i^	0.85 (1)	2.06 (1)	2.9034 (19)	173 (2)
O2—H2′⋯N1	0.84 (1)	1.89 (1)	2.6736 (15)	155 (2)
N2—H2⋯O3^ii^	0.87 (1)	2.06 (1)	2.8965 (17)	161 (2)
C9—H9*A*⋯*Cg* ^iii^	0.97	2.75	3.5896 (19)	145

Symmetry codes: (i) 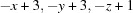; (ii) 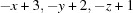; (iii) 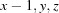.

**Fig. 1.**
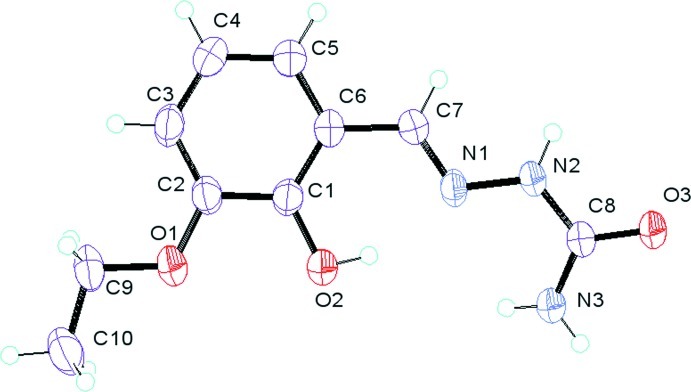
ORTEP view of the compound, drawn with 50% probability displacement ellipsoids for the non-H atoms.

**Fig. 2.**
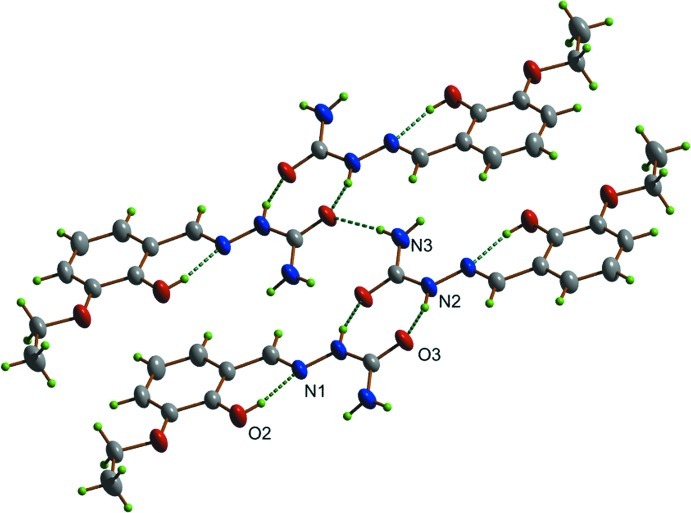
Graphical representation showing 1-D hydrogen bonding chain in the crystal structure of C10H13N3O3.

**Fig. 3.**
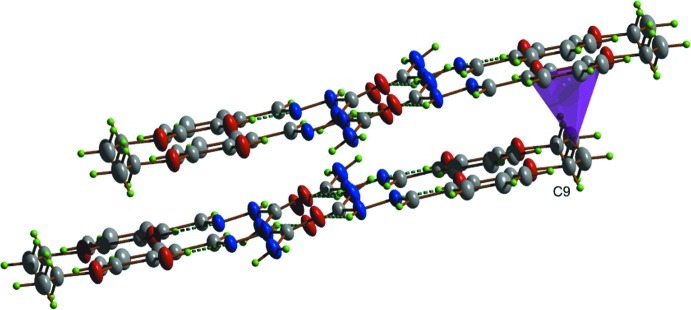
C–H···π interaction found in the title compound showing the linkage between layers.

**Fig. 4.**
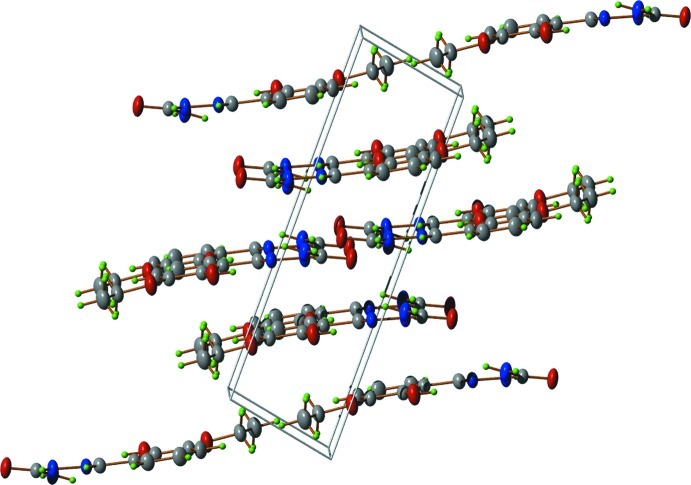
A view of the unit cell along b axis.

## supplementary crystallographic information

### Comment

The importance of semicarbazones lies in its pharmacological activities such as antitumoral (Afrasiabi *et al.*, 2005), antimicrobial (Siji *et al.*, 2010), antihypertensive, hypolipidemic, antineoplastic, hypnotic and anticonvulsant properties (Beraldo & Gambino, 2004). As the literature reports, the title compound, C_10_H_13_N_3_O_3_, is a tridentate semicarbazone ligand which formed complexes with vanadium (Noblía *et al.*, 2004, 2005; Rivadeneira *et al.*, 2009; Benítez *et al.*, 2009; Benítez *et al.*, 2011), and gallium (Gambino *et al.*, 2011) and have demonstrated to possess biological activity as antitumor and antiparasitic agents.

The compound crystallizes in triclinic, *P*1 space group. The molecule exists in the *E* configuration with respect to C7═N1 bond (Sithambaresan & Kurup, 2011) which is confirmed by the torsion angle of -176.32 (13)° of C6—C7—N1—N2 moiety (Fig. 1). The torsion angle value of -169.81 (13)° corresponding to N1—N2—C8—O3 moiety supports the *trans* configuration of the O3 atom with respect to the hydrazine nitrogen atom N1 similar to its phenyl derivative (Sithambaresan & Kurup, 2011). The torsion angle value of 10.5 (2)° corresponding to N1—N2—C8—N3 moiety supports the *cis* configuration of the N3 atom with respect to the nitrogen atom N1. Also the torsion angles of -1.8 (2)° and 6.9 (2)° for O2—C1—C6—C7 and C1—C6—C7—N1 moieties respectively confirm the *cis* configuration of phenolic oxygen O2 and azomethine nitrogen N1 and it favours intramolecular hydrogen bonding between N1 and H attached to O2. The molecule as a whole slightly goes out of planarity with maximum mean plane deviations of 0.392 (2)° at N(3) and -0.345 (1)° at O(3).

Even though atom O1 lies *cis* to O2, with a torsion angle of -0.8 (2)° (O2—C1—C2—O1) and N1 lies *cis* to N3, with a torsion angle of 10.5 (2)° (N1—N2—C8—N3), there are no intramolecular hydrogen bonding interactions involving N3—H3'···N1 and O2—H2'···O1 bonds, which makes the title compound different from its phenyl derivative (Sithambaresan & Kurup, 2011). The C7═N1 [1.278 (2) Å] and C8═O3 [1.2481 (17) Å] bond distances are very close to the formal C═N and C═O bond lengths [C═ N; 1.28 Å and C═O; 1.21 Å] (Allen *et al.*, 1987) respectively confirming the azomethine bond formation and the existence of semicarbazone in amido form in solid state. The N1—N2 [1.3749 (17) Å] and C8—N2 [1.352 (2) Å] bond distances lie in between the ideal values of corresponding single and double bonds [N—N; 1.45 and C—N; 1.47, N═N; 1.25 and C═N; 1.28] (Kala *et al.*, 2007) and it clearly proves the extended conjugation in the molecule.

Two conventional intermolecular hydrogen bonds are present in the molecular system (Fig. 2) between the O3 and the H atoms attached to N2 and N3 atoms of the neighbouring molecules with D···A distances of 2.8963 (19) and 2.9032 (18) Å. N2–H2···O3 hydrogen bonds form centrosymmetric dimers and these dimers are connected together by means of N3–H3A···O3 hydrogen bond to construct a 1-D hydrogen bonding chain and such chains are beautifully connected one over the other by C–H···π interaction (Fig. 3) with H···π distance of 2.7500 Å keeping the molecular system stable. Fig. 4 shows the packing diagram of the title compound along *b* axis.

### Experimental

The title compound was prepared by adapting a reported procedure (Sreekanth *et al.*, 2004). To a warm methanolic solution of hydrazinecarboxamide (0.1115 g, 1 mmol), a methanolic solution of 3-ethoxy-2-hydroxybenzaldehyde (0.1662 g, 1 mmol) was added and the resulting solution was refluxed for 6 h after adding 3 drops of conc. HCl. On cooling the solution, colorless crystals were separated out. Single crystals suitable for X-ray diffraction studies were obtained by slow evaporation of its solution in 1:1 mixture of methanol and DMF.

### Refinement

All H atoms on C were placed in calculated positions, guided by difference maps, with C–H bond distances 0.93–0.97 Å. H atoms were assigned as *U*_iso_=1.2Ueq (1.5 for Me). N2–H2 and O2–H2' H atoms were located from difference maps and restrained using *DFIX* instructions. N3–H3A and N3–H3B H atoms were also located from difference maps and restrained using *DFIX* and DANG instructions. Omitted owing to bad disagreement was the reflection (0 0 1).

### Crystal data

**Table:**

C_10_H_13_N_3_O_3_	*Z* = 2
*M**_r_* = 223.23	*F*(000) = 236.0
Triclinic, *P*1	*D*_x_ = 1.403 Mg m^−^^3^
Hall symbol: -P 1	Mo *K*α radiation, λ = 0.71073 Å
*a* = 5.0676 (4) Å	Cell parameters from 1468 reflections
*b* = 7.0426 (7) Å	θ = 3.1–27.8°
*c* = 15.8394 (15) Å	µ = 0.11 mm^−^^1^
α = 97.509 (4)°	*T* = 296 K
β = 98.819 (3)°	Needle, colorless
γ = 105.790 (4)°	0.30 × 0.25 × 0.20 mm
*V* = 528.62 (8) Å^3^	

### Data collection

**Table:**

Bruker Kappa APEXII CCD diffractometer	1794 independent reflections
Radiation source: fine-focus sealed tube	1496 reflections with *I* > 2σ(*I*)
Graphite monochromator	*R*_int_ = 0.011
Detector resolution: 8.33 pixels mm^-1^	θ_max_ = 25.0°, θ_min_ = 2.7°
ω and φ scan	*h* = −4→6
Absorption correction: multi-scan (*SADABS*; Bruker, 2004)	*k* = −8→7
*T*_min_ = 0.969, *T*_max_ = 0.979	*l* = −18→18
2559 measured reflections	

### Refinement

**Table:**

Refinement on *F*^2^	Secondary atom site location: difference Fourier map
Least-squares matrix: full	Hydrogen site location: inferred from neighbouring sites
*R*[*F*^2^ > 2σ(*F*^2^)] = 0.037	H atoms treated by a mixture of independent and constrained refinement
*wR*(*F*^2^) = 0.109	*w* = 1/[σ^2^(*F*_o_^2^) + (0.0603*P*)^2^ + 0.0879*P*] where *P* = (*F*_o_^2^ + 2*F*_c_^2^)/3
*S* = 1.05	(Δ/σ)_max_ < 0.001
1794 reflections	Δρ_max_ = 0.15 e Å^−^^3^
163 parameters	Δρ_min_ = −0.18 e Å^−^^3^
5 restraints	Extinction correction: *SHELXL97* (Sheldrick, 2008), Fc^*^=kFc[1+0.001xFc^2^λ^3^/sin(2θ)]^-1/4^
Primary atom site location: structure-invariant direct methods	Extinction coefficient: 0.043 (11)

### Special details

**Table:**

Geometry. All e.s.d.'s (except the e.s.d. in the dihedral angle between two l.s. planes) are estimated using the full covariance matrix. The cell e.s.d.'s are taken into account individually in the estimation of e.s.d.'s in distances, angles and torsion angles; correlations between e.s.d.'s in cell parameters are only used when they are defined by crystal symmetry. An approximate (isotropic) treatment of cell e.s.d.'s is used for estimating e.s.d.'s involving l.s. planes.
Refinement. Refinement of *F*^2^ against ALL reflections. The weighted *R*-factor *wR* and goodness of fit *S* are based on *F*^2^, conventional *R*-factors *R* are based on *F*, with *F* set to zero for negative *F*^2^. The threshold expression of *F*^2^ > σ(*F*^2^) is used only for calculating *R*-factors(gt) *etc*. and is not relevant to the choice of reflections for refinement. *R*-factors based on *F*^2^ are statistically about twice as large as those based on *F*, and *R*- factors based on ALL data will be even larger.

### Fractional atomic coordinates and isotropic or equivalent isotropic displacement parameters (Å^2^)

**Table:**

	*x*	*y*	*z*	*U*_iso_*/*U*_eq_	
O1	0.0222 (2)	0.77360 (18)	0.14679 (8)	0.0535 (4)	
O2	0.4871 (2)	0.95529 (17)	0.25818 (8)	0.0507 (4)	
N3	1.1509 (3)	1.3215 (2)	0.44231 (10)	0.0451 (4)	
N1	0.9230 (2)	0.92842 (19)	0.36957 (8)	0.0378 (3)	
N2	1.1846 (3)	1.0000 (2)	0.42199 (9)	0.0415 (4)	
O3	1.5573 (2)	1.25778 (16)	0.48904 (8)	0.0484 (3)	
C1	0.4059 (3)	0.7519 (2)	0.24373 (10)	0.0380 (4)	
C2	0.1566 (3)	0.6509 (2)	0.18340 (10)	0.0409 (4)	
C3	0.0673 (3)	0.4439 (3)	0.16548 (11)	0.0482 (4)	
H3	−0.0981	0.3773	0.1258	0.058*	
C4	0.2212 (4)	0.3340 (3)	0.20585 (12)	0.0518 (5)	
H4	0.1593	0.1945	0.1932	0.062*	
C5	0.4660 (3)	0.4317 (3)	0.26476 (11)	0.0460 (4)	
H5	0.5691	0.3576	0.2916	0.055*	
C6	0.5613 (3)	0.6415 (2)	0.28465 (9)	0.0374 (4)	
C7	0.8283 (3)	0.7384 (2)	0.34449 (9)	0.0383 (4)	
H7	0.9336	0.6587	0.3652	0.046*	
C8	1.3056 (3)	1.1985 (2)	0.45241 (9)	0.0359 (4)	
C9	−0.2192 (3)	0.6811 (3)	0.07918 (11)	0.0510 (5)	
H9A	−0.3608	0.5872	0.1005	0.061*	
H9B	−0.1694	0.6088	0.0307	0.061*	
C10	−0.3277 (4)	0.8456 (3)	0.05117 (13)	0.0641 (6)	
H10A	−0.3775	0.9153	0.0996	0.096*	
H10B	−0.4897	0.7890	0.0054	0.096*	
H10C	−0.1854	0.9378	0.0305	0.096*	
H2	1.274 (3)	0.917 (2)	0.4371 (11)	0.045 (5)*	
H2'	0.642 (3)	0.984 (3)	0.2920 (12)	0.079 (7)*	
H3A	1.223 (3)	1.4467 (15)	0.4605 (12)	0.061 (6)*	
H3B	0.975 (2)	1.278 (3)	0.4271 (13)	0.068 (6)*	

### Atomic displacement parameters (Å^2^)

**Table:**

	*U*^11^	*U*^22^	*U*^33^	*U*^12^	*U*^13^	*U*^23^
O1	0.0390 (6)	0.0514 (8)	0.0589 (7)	0.0127 (5)	−0.0154 (5)	0.0016 (6)
O2	0.0410 (7)	0.0387 (7)	0.0609 (8)	0.0109 (5)	−0.0143 (5)	−0.0013 (5)
N3	0.0284 (7)	0.0373 (8)	0.0621 (9)	0.0075 (6)	−0.0051 (6)	0.0045 (7)
N1	0.0270 (7)	0.0419 (8)	0.0375 (7)	0.0069 (5)	−0.0039 (5)	0.0016 (6)
N2	0.0284 (7)	0.0392 (8)	0.0493 (8)	0.0102 (6)	−0.0094 (5)	0.0010 (6)
O3	0.0246 (6)	0.0407 (7)	0.0695 (8)	0.0063 (5)	−0.0086 (5)	0.0021 (5)
C1	0.0323 (8)	0.0383 (9)	0.0383 (8)	0.0081 (7)	0.0016 (6)	0.0009 (6)
C2	0.0315 (8)	0.0461 (10)	0.0405 (8)	0.0107 (7)	−0.0008 (6)	0.0029 (7)
C3	0.0345 (9)	0.0492 (10)	0.0474 (9)	0.0026 (7)	−0.0057 (7)	−0.0013 (7)
C4	0.0468 (10)	0.0385 (9)	0.0569 (10)	0.0015 (8)	−0.0026 (8)	0.0018 (8)
C5	0.0434 (9)	0.0411 (9)	0.0471 (9)	0.0086 (7)	−0.0022 (7)	0.0075 (7)
C6	0.0316 (8)	0.0418 (9)	0.0341 (8)	0.0075 (7)	0.0018 (6)	0.0032 (6)
C7	0.0341 (8)	0.0392 (9)	0.0381 (8)	0.0108 (7)	−0.0012 (6)	0.0050 (7)
C8	0.0270 (7)	0.0386 (8)	0.0382 (8)	0.0075 (6)	0.0006 (6)	0.0053 (6)
C9	0.0367 (9)	0.0647 (12)	0.0425 (9)	0.0128 (8)	−0.0072 (7)	−0.0004 (8)
C10	0.0483 (11)	0.0769 (14)	0.0604 (12)	0.0167 (10)	−0.0099 (9)	0.0168 (10)

### Geometric parameters (Å, º)

**Table:**

O1—C2	1.3697 (19)	C3—C4	1.386 (2)
O1—C9	1.4319 (18)	C3—H3	0.9300
O2—C1	1.3558 (19)	C4—C5	1.377 (2)
O2—H2'	0.837 (10)	C4—H4	0.9300
N3—C8	1.326 (2)	C5—C6	1.400 (2)
N3—H3A	0.849 (9)	C5—H5	0.9300
N3—H3B	0.846 (9)	C6—C7	1.459 (2)
N1—C7	1.278 (2)	C7—H7	0.9300
N1—N2	1.3749 (17)	C9—C10	1.499 (3)
N2—C8	1.352 (2)	C9—H9A	0.9700
N2—H2	0.869 (9)	C9—H9B	0.9700
O3—C8	1.2481 (17)	C10—H10A	0.9600
C1—C6	1.397 (2)	C10—H10B	0.9600
C1—C2	1.407 (2)	C10—H10C	0.9600
C2—C3	1.380 (2)		
			
C2—O1—C9	117.61 (13)	C6—C5—H5	119.7
C1—O2—H2'	102.2 (16)	C1—C6—C5	119.31 (14)
C8—N3—H3A	120.2 (13)	C1—C6—C7	121.94 (14)
C8—N3—H3B	121.7 (13)	C5—C6—C7	118.68 (14)
H3A—N3—H3B	117.1 (17)	N1—C7—C6	122.59 (14)
C7—N1—N2	116.34 (13)	N1—C7—H7	118.7
C8—N2—N1	121.45 (13)	C6—C7—H7	118.7
C8—N2—H2	118.6 (12)	O3—C8—N3	122.89 (15)
N1—N2—H2	120.0 (12)	O3—C8—N2	118.66 (13)
O2—C1—C6	122.85 (14)	N3—C8—N2	118.45 (13)
O2—C1—C2	117.48 (14)	O1—C9—C10	107.13 (15)
C6—C1—C2	119.66 (15)	O1—C9—H9A	110.3
O1—C2—C3	125.56 (14)	C10—C9—H9A	110.3
O1—C2—C1	114.78 (14)	O1—C9—H9B	110.3
C3—C2—C1	119.66 (14)	C10—C9—H9B	110.3
C2—C3—C4	120.82 (15)	H9A—C9—H9B	108.5
C2—C3—H3	119.6	C9—C10—H10A	109.5
C4—C3—H3	119.6	C9—C10—H10B	109.5
C5—C4—C3	119.85 (16)	H10A—C10—H10B	109.5
C5—C4—H4	120.1	C9—C10—H10C	109.5
C3—C4—H4	120.1	H10A—C10—H10C	109.5
C4—C5—C6	120.70 (15)	H10B—C10—H10C	109.5
C4—C5—H5	119.7		
			
C7—N1—N2—C8	−179.98 (14)	C2—C1—C6—C5	0.1 (2)
C9—O1—C2—C3	−5.3 (2)	O2—C1—C6—C7	−1.8 (2)
C9—O1—C2—C1	174.53 (14)	C2—C1—C6—C7	176.94 (13)
O2—C1—C2—O1	−0.8 (2)	C4—C5—C6—C1	−0.3 (3)
C6—C1—C2—O1	−179.55 (13)	C4—C5—C6—C7	−177.26 (14)
O2—C1—C2—C3	179.03 (14)	N2—N1—C7—C6	−176.32 (13)
C6—C1—C2—C3	0.3 (2)	C1—C6—C7—N1	6.9 (2)
O1—C2—C3—C4	179.43 (16)	C5—C6—C7—N1	−176.21 (15)
C1—C2—C3—C4	−0.4 (3)	N1—N2—C8—O3	−169.81 (13)
C2—C3—C4—C5	0.1 (3)	N1—N2—C8—N3	10.5 (2)
C3—C4—C5—C6	0.2 (3)	C2—O1—C9—C10	179.40 (14)
O2—C1—C6—C5	−178.64 (14)		

### Hydrogen-bond geometry (Å, º)

Cg is the centroid of the C1–C6 ring

**Table:**

*D*—H···*A*	*D*—H	H···*A*	*D*···*A*	*D*—H···*A*
N3—H3*A*···O3^i^	0.85 (1)	2.06 (1)	2.9034 (19)	173 (2)
O2—H2′···N1	0.84 (1)	1.89 (1)	2.6736 (15)	155 (2)
N2—H2···O3^ii^	0.87 (1)	2.06 (1)	2.8965 (17)	161 (2)
C9—H9*A*···*Cg*^iii^	0.97	2.75	3.5896 (19)	145

Symmetry codes: (i) −*x*+3, −*y*+3, −*z*+1; (ii) −*x*+3, −*y*+2, −*z*+1; (iii) *x*−1, *y*, *z*.
